# Impact of electric vehicle charging demand on power distribution grid congestion

**DOI:** 10.1073/pnas.2317599121

**Published:** 2024-04-22

**Authors:** Yanning Li, Alan Jenn

**Affiliations:** ^a^Civil and Environmental Engineering, Institute of Transportation Studies, University of California, Davis, CA 95616

**Keywords:** transport electrification, electric vehicles, energy policy, grid reinforcements, distribution system

## Abstract

California, a pioneer in EV adoption, has enacted ambitious electric vehicle (EV) policies that will generate a large burden on the state’s electric distribution system. We investigate the statewide impact of uncontrolled EV charging on the electric distribution networks at a large scale and high granularity, by employing an EV charging profile projection that combines travel demand model, EV adoption model, and real-world EV charging data. We find a substantial need for infrastructure upgrades in 50% of feeders by 2035, and 67% of feeders by 2045. The distribution system across California must upgrade its capacity by 25 GW by 2045, corresponding to a cost between $6 and $20 billion. While the additional infrastructure cost drives the electricity price up, it is offset by the downward pressure from the growth of total electricity consumption and leads to a reduction in electricity rate between $0.01 and $0.06/kWh by 2045. We also find that overloading conditions are highly diverse spatially, with feeders in residential areas requiring twice as much upgrade compared to commercial areas. Our study provides a framework for evaluating EVs’ impact on the distribution grid and indicates the potential to reduce infrastructure upgrade costs by shifting home-charging demand. The imminent challenges confronting California serve as a microcosm of the forthcoming obstacles anticipated worldwide due to the prevailing global trend of EV adoption.

## Significance

This study conducts a quantification of electric vehicles’ (EVs) impact on distribution grids—the primary bottleneck of EV-grid integration. We find that 67% of the feeders in California will need capacity upgrades by 2045, with a total of 25 GW upgrades needed, corresponding to a cost between $6 and $20 billion. However, the effect of the additional cost is offset by downward pressure on electricity rates due to the overall growth in electricity consumption, leading to an overall rate reduction between $0.01 and $0.06/kWh. Our work indicates that feeders in residential areas will require twice as much upgrade compared to commercial areas, revealing the potential to ease the grid’s stress by shifting home-charging demand.

Electric vehicles (EVs) are rapidly being adopted to help decarbonize the transportation sector. The International Energy Agency (IEA) reports a substantial surge in global EV stock, surpassing 10 million over the last decade, with projections estimating an escalation up to 300 million by 2030 (1). California is a crucial pioneer in EV adoption and has committed to reducing greenhouse gas (GHG) emissions in the transportation sector by establishing ambitious policies to foster EV sales growth. The state has a goal of 5 million EVs on the road in California by 2030, and recent updates to the Zero Emissions Vehicle rule sets requirements for 100% sales of new passenger vehicles to be electric by 2035 (2). This widespread adoption of EVs in the future will lead to a large growth in electricity charging load, which will contribute to challenges in the operation and planning of the power system. Exploring these challenges within the context of California can provide insights that extend beyond its borders, highlighting upcoming hurdles from the global trend of EV adoption.

The power system consists of electricity generation, transmission, and distribution systems. A vast body of literature has investigated the integration of EVs into the existing power grids, though most of these studies focus on the bulk power level (generation and transmission) (3–7), i.e., how generation dispatch and transmission congestion will be affected by the increasing EV charging load, and how managing EV charging accordingly can help enhance grid stability (3, 7) and renewable integration (5, 6). However, managing EV charging without accounting for distribution constraints may result in even more severe congestion compared to unmanaged charging (8). Since EV charging predominantly occurs within the distribution grid, if the distribution capacity remains unprepared or insufficiently upgraded, it could become the primary bottleneck of EV penetration into the grid, risking the power quality and reliability of the local network (9). Therefore, it is imperative to understand how constrained the distribution grid will be and what upgrades are needed for the integration of future EV charging demand into the distribution system.

Studies that examine EV charging demand in the distribution grid usually focus on substations and/or feeders. Substations are the connections that step down the high-voltage electricity from the transmission grid to lower voltages for local power distribution. Feeders, also called circuits, often refer to the conductors and transformers that deliver the stepped-down electricity to end-use consumers. Infrastructure specifications and electricity usage patterns are highly diverse at this level of resolution, so the results of these studies are highly varied due to the heterogeneity in the characteristics of the chosen distribution networks. However, most of the feeder-level studies use hypothetical network models rather than real-world networks (9–15), which provide limited implication for real-world infrastructure upgrade needs. Among the studies that do employ real-world distribution network data, many are constrained in scope, covering only a single feeder (16, 17), a single workplace (18), or a single distribution network with one substation and several feeders (19–23). Only a handful of studies cover distribution systems with multiple substations (8, 10, 24–28). It is a challenge to capture the spatial heterogeneity of large-scale systems at the feeder level due to data availability and computational complexity. Crozier et al. (29) studied the distribution system of the whole Great Britain, but the simulation is based on 3 typical network models. Jenn and Highleyman (30) and Elmallah et al. (31) focused on the feeders in the territory of Pacific Gas & Electric (PG&E), which consists of around 700 substations. In this study, we cover the entire distribution system in the territories of all three major investor-owned-utilities (IOUs) of California: PG&E, serving the majority of northern California and approximately 40% of the state’s population; Southern California Edison (SCE), covering the greater Los Angeles area and serving around 38% of the population; and San Diego Gas & Electric (SDG&E), covering the greater San Diego area and serving about 10% of the population. This extensive scale includes a total of over 1,600 substations and over 5,000 feeders. Similar coverage has only been reported by the California Public Utilities Commission (CPUC) (32, 33), but their analysis simplifies the representation of EV charging demand.

A major challenge of simulating the real-world impact of future EVs on the distribution system is the spatial and temporal diversities in charging loads. For light-duty EVs, this diversity is the culmination of the complex interplay between EV travel behavior, EV charging decision, and EV adoption. Top–down approaches (31, 33) are limited in their ability to capture all these aspects of variabilities. Among the bottom–up approaches, many simulate the travel and charging behavior of EVs simultaneously. The simplest method is to sample these behaviors randomly from probability distribution functions (10, 15, 20), which models the variability but can deviate from reality. Another common means is to use data records from EV pilot projects (18, 21, 23, 25, 30), which works well for small-scale analysis, but can be biased samples for large-scale studies. Many other studies model the travel and charging behaviors separately. To simulate EV travel demand, conventional vehicle travel surveys are usually adopted (8, 13, 17, 19, 24, 27–29, 32, 34), which are more comprehensive and cover the locational variation of travel behavior. In this study, we utilize the California statewide travel demand model (CSTDM) (35), which provides high-resolution conventional vehicle flows categorized by trip purposes.

When it comes to modeling the EV charging behavior, many studies make a strong assumption that all EVs are involved in charging management, or smart charging (8, 13, 15, 18, 21, 23, 27–29, 36). While managed (“smart”) charging is a potential “ideal” solution for many grid issues, studying unmanaged charging is a necessary first step to examine grid impacts in the absence of interventions, and to understand toward what directions should we manage charging behavior. A common way to represent unmanaged charging is assuming that EVs would start charging immediately after arrival (8, 10, 11, 19, 20, 24, 27, 32). However, real-world EV charging behavior can be substantially different (30)—a shortcoming in the literature as very few studies combine conventional vehicle travel data with empirical EV charging records to model EV charging behavior (17, 29, 34). We employ empirical EV charging data from the utilities, public-charging service providers, and data logger records. Combined with the travel behavior data, we can tackle the diversity in EV charging behavior across a variety of locations.

Capturing the regional variation of EV adoption is also crucial when conducting analysis on a large scale. The most common approach is to allocate EV penetration according to the spatial distribution of household (24) or conventional vehicle ownership (25, 27, 28). Few studies fit EV growth models for different regions separately (29, 30, 32). In this work, we spatially differentiate EV penetration at high-resolution employing a model that projects EV adoption with multiple social-economic factors at household level—the EV Toolbox (37), while calibrating the model’s aggregate output to align with California’s regulatory requirements.

**Fig. 1.**
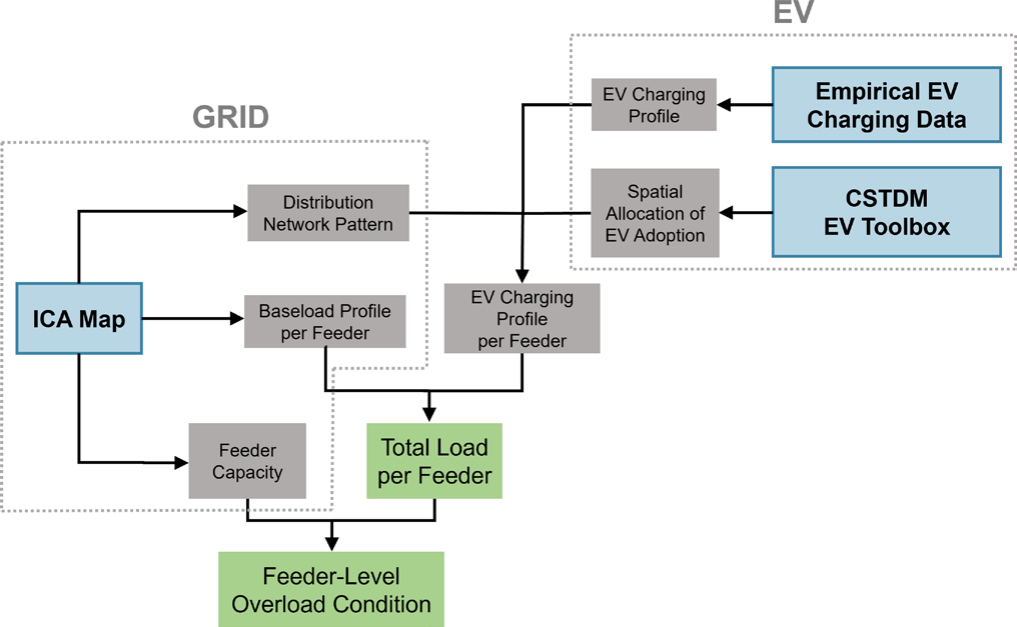
Data sources (blue), intermediate data (gray), and outputs (green) in the general research framework. Spatial and temporal data at feeder level from both the grid and the EV side are utilized to perform a bottom–up projection on the hourly EV charging load and baseload profile by feeder. On the grid side, we adopt feeder-level capacity and baseload data from the Integration Capacity Analysis (ICA) map. For EVs, we combine the CSTDM, EV adoption model - EV Toolbox, and empirical EV charging data to project future EV charging profiles at high granularity and large scale.

As shown in Fig. 1, a distinct aspect of our work is the combination of travel demand model, empirical EV charging data, and EV adoption model to simulate uncontrolled EV charging profiles at high resolution and large scale. By mapping them to the distribution feeders, we can analyze the statewide development of feeder overloads caused by EV uptake, as well as the corresponding circuit upgrade capacity need and costs generated. The remainder of the paper is organized as follows: we first present the statewide development of feeder overloads, circuit upgrade needs, and corresponding costs on an aggregate level. Next, we discuss how the different types of charging locations affect feeder overloading. Then we cover the nuances of what determines the overload timing. Finally, we conclude with a discussion on the major implications and outlook of our work.

## Results

### Distribution Feeder Overloads and Upgrades.

**Fig. 2.**
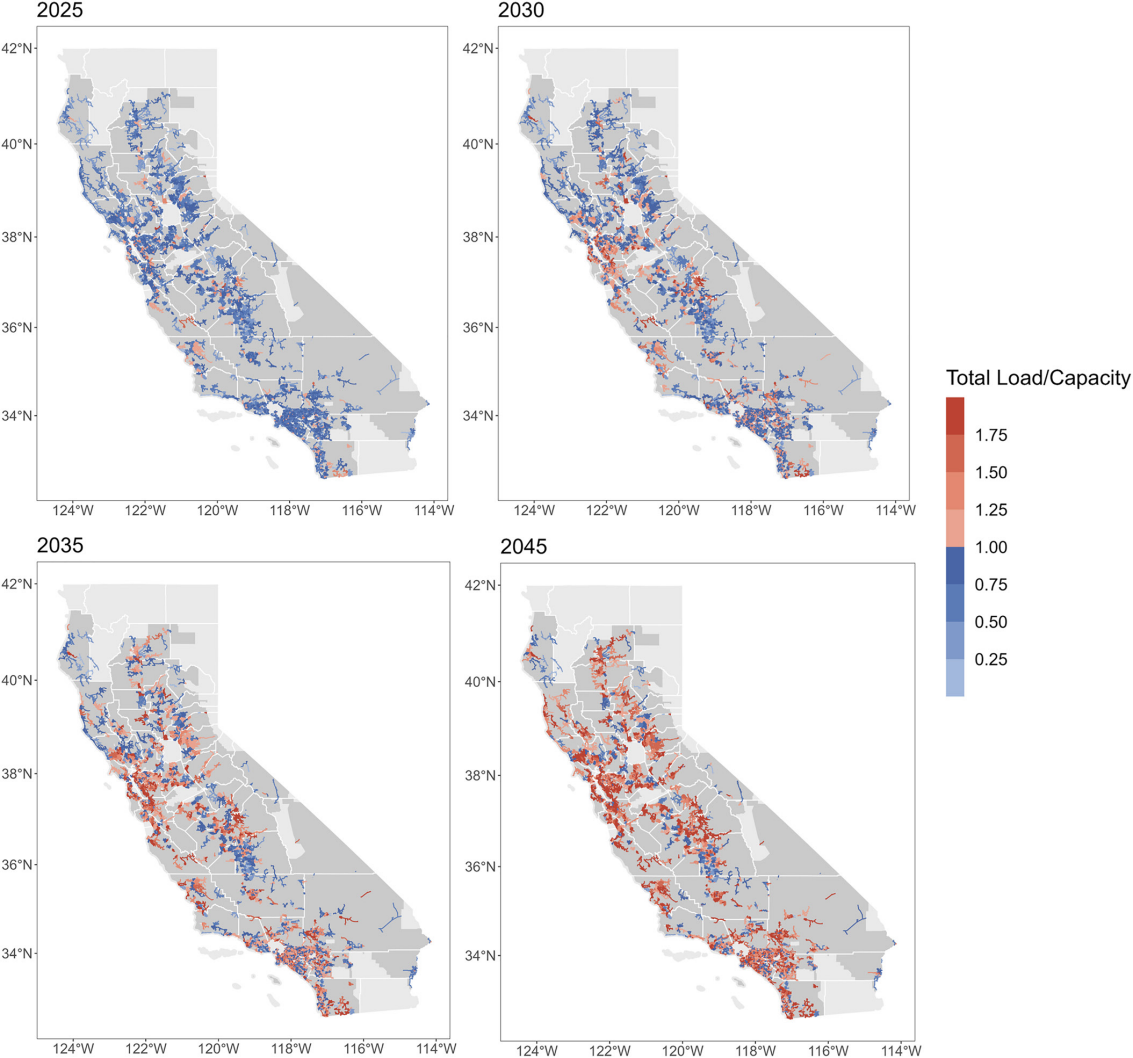
Spatial distribution of peak load intensity of the feeders in different years. The feeders are colored by the ratio of total peak load (baseload and EV charging load) over capacity. Feeders colored red are overloaded, and those colored blue are not overloaded (including the feeders with peak total load equals capacity). The darker the color, the more severe the overloading, or the closer to overloading if not already overloaded. Areas in darker gray are the territories of the three major IOUs in California.

By 2045, most feeders in California need to be upgraded. These infrastructure upgrades depend on the extent the peak load on each feeder exceeds the capacity limit threshold. In Fig. 2, we show how feeders become increasingly stressed over time due to the growing EV charging demand. These overloads (colored red) generally start to appear in the population-dense areas such as the Bay Area, but are mostly less intense in the early 2020s—with the overload power below 25% of the existing feeder capacity. The early-overloaded feeders continue to become intensely stressed with the growth of EV charging demand while more feeders become overloaded with the expansion of EV uptake. By 2045, most feeders are severely loaded with the total load reaching nearly twice the current capacity. Neighboring feeders that are not yet overloaded (colored blue) tends to be very close to overloading as well—with less than 25% of capacity headroom remained.

**Fig. 3.**
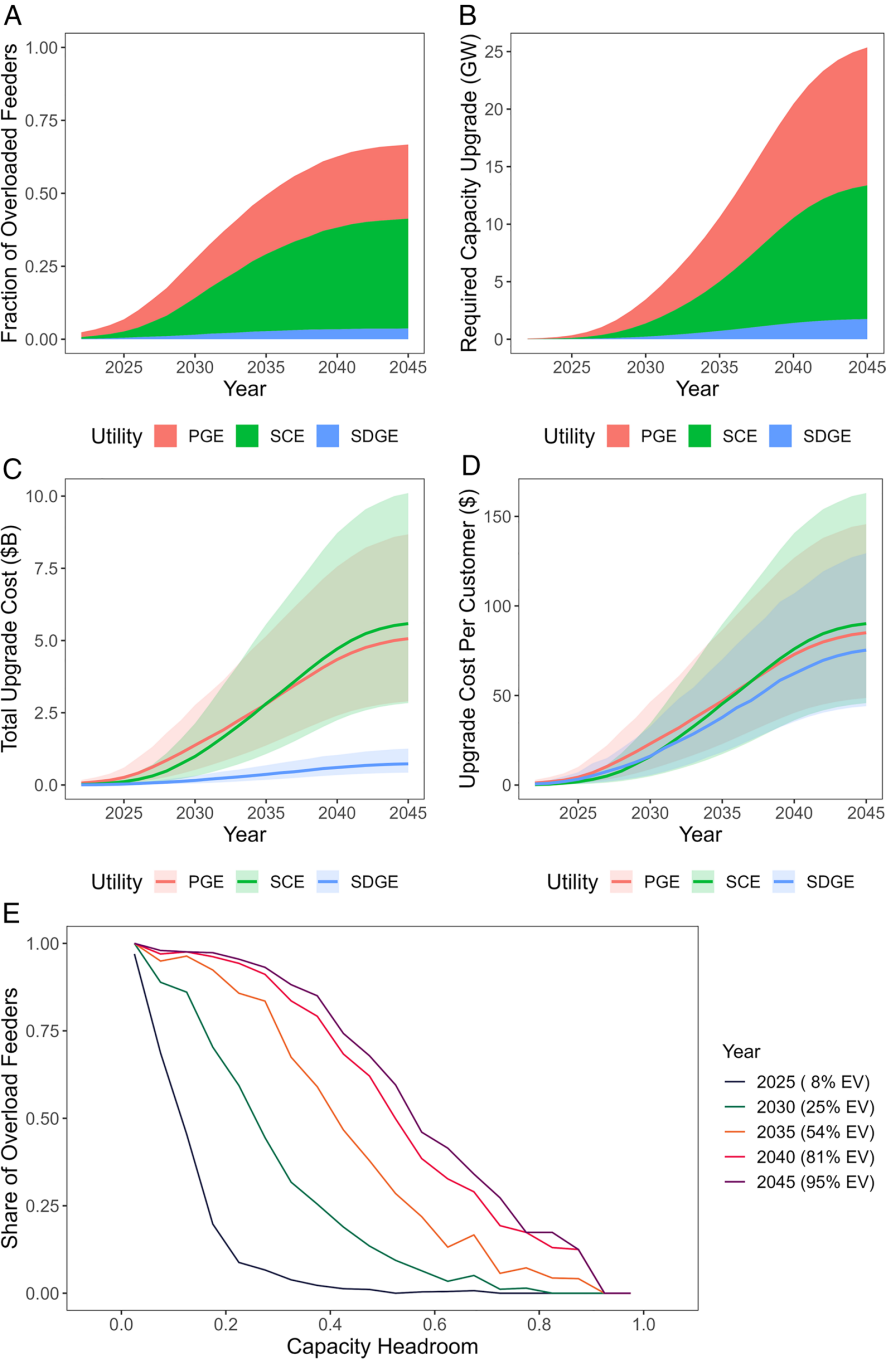
The growth of distribution grid needs in CA utility territories over time. (*A*) The share of feeders that are overloaded by EV charging demand. (*B*) Total feeder upgrade capacity needed, estimated by adding up the maximum overload power on every feeder. (*C*) Total feeder upgrade cost, where the lines are calculated using the median of reported costs, and the upper and lower edges of the ribbons are calculated with the 75th and 25th percentile of reported costs. (*D*) Total feeder upgrade cost per customer. (*E*) Given the feeder capacity headroom left in baseline year 2022, the share of overloaded feeders across the feeder population with the same headroom.

The growth of overloaded distribution infrastructure with EV uptake over the years is shown in Fig. 3. The number of overload feeders (Fig. 3*A*) ramp up rapidly from mid-2020s to mid-2030s, with only 7% of feeders overloaded by EVs in 2025, but that grows to a total of 27% in 2030 and 50% in 2035. The growth slows down afterward, rising to 63% in 2040 and up to 67% in 2045. PG&E takes up most of the overloaded feeders in the 2020s, but SCE catches up after 2030. The total upgrade need (Fig. 3*B*) grows dramatically from 3.5 GW in 2030 to 25.4 GW in 2045. Although SCE has more feeders that need to be upgraded after 2030, PG&E has the highest requirements for capacity upgrades. This indicates that while the SCE territory has the widest range of overloading, the magnitude of overloading in the PG&E territory is generally the most severe.

The range of corresponding infrastructure upgrade costs is estimated using the 25th, 50th, and 75th percentiles of reported circuit upgrade cost per kW at different scales (*Methods*, *Distribution Grid Data*), and depicted in Fig. 3*C*. The range of circuit upgrade costs is quite broad, with the total cost by 2045 ranging from $6 billion to $20 billion. The total historical distribution grid capital cost of the 3 IOUs through 2022 is around $51 billion in total, with a split of around $18 billion from PG&E, $27 billion from SCE, and $6 billion from SDG&E (38). And the total operation and maintenance cost in 2022 is around $6 billion (38). The statewide EV-related upgrade cost in the next two decades is expected to be around 10 to 40% of the total existing distribution grid capital costs, or around 1 to 3 times the annual operation and maintenance costs. The increase in infrastructure costs puts an upward pressure on electricity rates, while the growth of EV load makes a downward pressure. Assuming a 7.5% rate of return (38) and considering only the distribution grid rate base, we estimate a negative net rate impact from EV growth for all three utilities: By 2045, the impact is around −0.006 $ kWh for PG&E, −0.011 $/kWh for SCE, and −0.062 $/kWh for SDG&E. This aligns with CPUC’s estimations (33). While electricity price may decrease, the overall bill of all consumers will still increase due to the growth of total electricity consumption, which will make up for the total upgrade costs. We note that the uncertainty could be even higher in actual future upgrade costs, since we are using near-term cost projections from the utilities and are not considering possible future change in the costs due to technology development or bottlenecks.

Among the three IOUs, PG&E accounts for the largest split of these costs in early years, and from 2035 SCE is estimated to need the highest upgrade costs. The difference in total upgrades and costs among different IOU territories are largely caused by the different numbers of customers they serve. As can be seen in Fig. 3*D*, the upgrade costs per customer in the three IOU territories are similar in scale, but the difference in their trends can still be observed. These differences are a strong indication that impacts to distribution grids are unlikely to be homogenous in other territories as well, and utilities may need to prepare for upgrades based on local conditions without relying on insights derived from other regions.

Despite these differences in the total upgrade needs and costs, some generalized trends can be observed in the relationship between capacity headroom left in each individual feeder and the possibility of overloading among the feeders with similar headroom, which is shown in Fig. 3*E*. In 2022, feeders with a headroom of over 50% of its capacity are safe from being overloaded by EV charging even with as high as 8% of EVs in total LDV stock. But when EV adoption grows to 25%, only the feeders with more than 80% headroom currently will be all safe. As EVs surpass 50% of the LDV population, only the feeders with over 90% headroom can completely avoid upgrade. But in general, the greater the headroom preserved in a feeder at present, the lower the possibility of requiring an upgrade in the future as the EV population expands. Even with a 95% EV adoption rate, among the feeders with over 55% headroom today, less than half of them will need an upgrade. Therefore, to reduce potential upgrade costs, a high-resolution forecast of EV charging load is essential to accurately determine which of the feeders are problematic.

### The Impact of Charging Location on Feeder Overload.

**Fig. 4.**
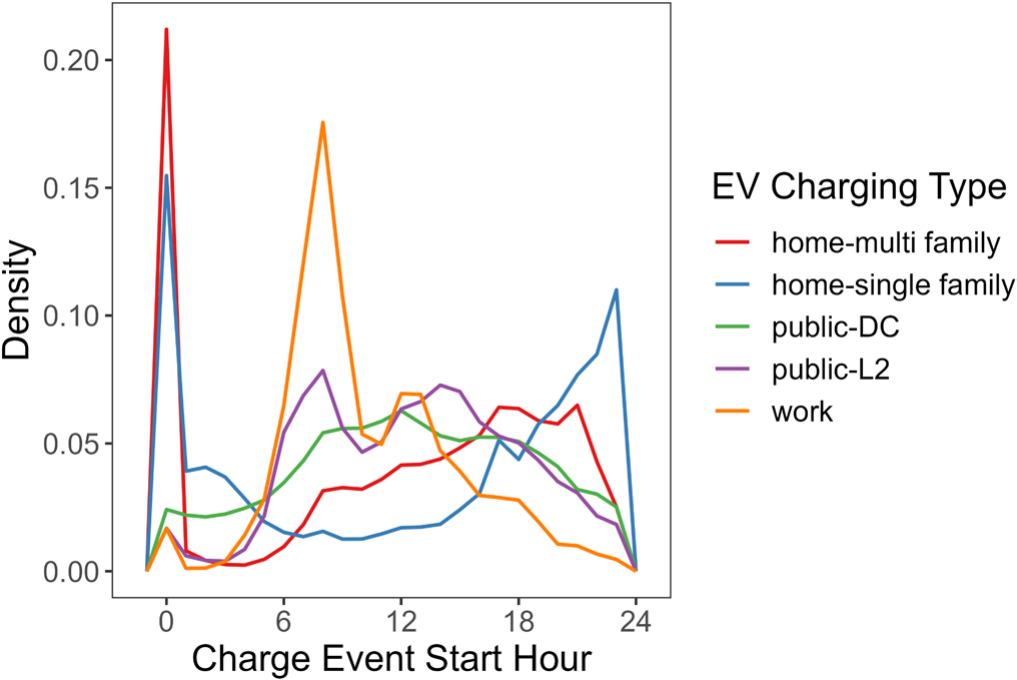
Distribution of charge event start hour of different types of charging. The data is from empirical EV charging session records.

EV charging patterns at different locations can vary significantly, as can be seen in Fig. 4. Workplace charging mostly start around 8:00 in the morning, when people arrive at work; and home charging usually start after 18:00 when EV owners get back; the start time of public charging tend to be distributed a bit more in the middle, when people are running errands. For home charging, chargers that are installed at multifamily dwellings have more events that start earlier in the day than those in single-family homes. And within public charging, L2 and DC fast charging events also have quite different distributions of charging behavior.

**Fig. 5.**
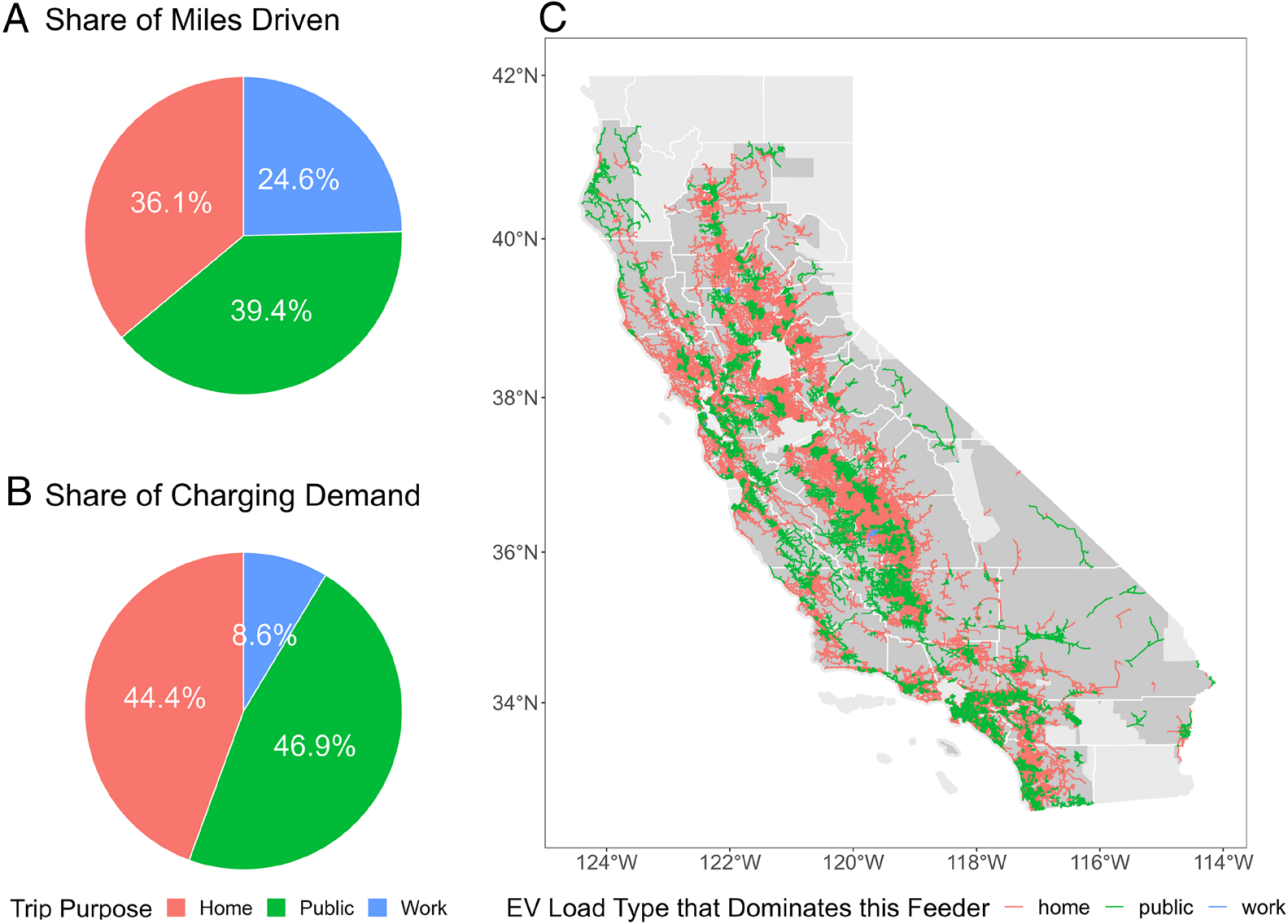
Distribution of travel and charging demand by trip purpose. (*A*) Share of total miles driven by LDVs in California among trips going home, to work, and to public locations. (*B*) Share of EV charging demand at different charging locations in 2045. (*C*) Spatial distribution of feeder categorization by the major EV load type that dominates on each feeder in 2045. 57.8% of the feeders are dominated by home charging load, 42.1% dominated by public charging, and 0.1% dominated by workplace charging.

The variation in electricity usage at different types of locations can have a strong impact on how the grid is strained by the load. Studies have shown that overnight home charging puts more burden on the bulk level of the power system than daytime workplace or public charging (6) but a similar comparison at the distribution level is still lacking. We simulate EV charging profiles by bootstrapping from empirical charging data (separately for each type of charging location), based on different trip purposes derived from a travel demand model. This allows us to observe the spatial distribution of different types of charging load and how they affect the local distribution network differently. Fig. 5*A* shows the split of total travel demand in California among trips going home, to work, or to public locations. Compared to the split of EV charging demand shown in Fig. 5*B*, it can be observed that the share of home- and public-charging demand is higher than that of travel demand. The reason is twofold: 1) People do not necessarily charge after each short-distance trip, and their choice of charging locations within a tour depends highly on their preference, which is modeled based on survey data in our analysis (*Materials and Methods*, *EV Charging Load Simulation*). According to the survey, the majority of people prefer to charge at home (39); 2) During long-distance trips, people may stop on the way to charge at public-charging stations, causing an increase in public-charging load.

We categorize the feeders by the charging load type that draws the most energy from each feeder. The map shown in Fig. 5*C* depicts the spatial distribution of the feeder categorization. The majority of feeders are dominated by either home-charging load or public-charging load. Very few feeders are dominated by workplace charging, due to both the relatively lower travel demand and the mostly lower power level of workplace chargers. These workplace charging feeders are never overloaded according to our results, so we only discuss the home- and public-charging feeders in the following sections. This implies a potential to reduce or even eliminate infrastructure congestion by encouraging workplace charging.

Home charging–dominated feeders are generally more stressed by the EV uptake than the public charging–dominated feeders. After 2025, the number of overloaded home-charging feeders are nearly twice as much the number of overloaded public-charging feeders. A similar ratio is estimated for the total capacity upgrade needs in home vs public-charging feeders. By 2045, home-charging feeders statewide will need around 16 GW capacity increase, with a corresponding cost of $3.9 billion to $12.9 billion; while public-charging feeders need 9 GW of upgrade, with a corresponding cost ranging from $2.2 billion to $7.1 billion.

**Fig. 6.**
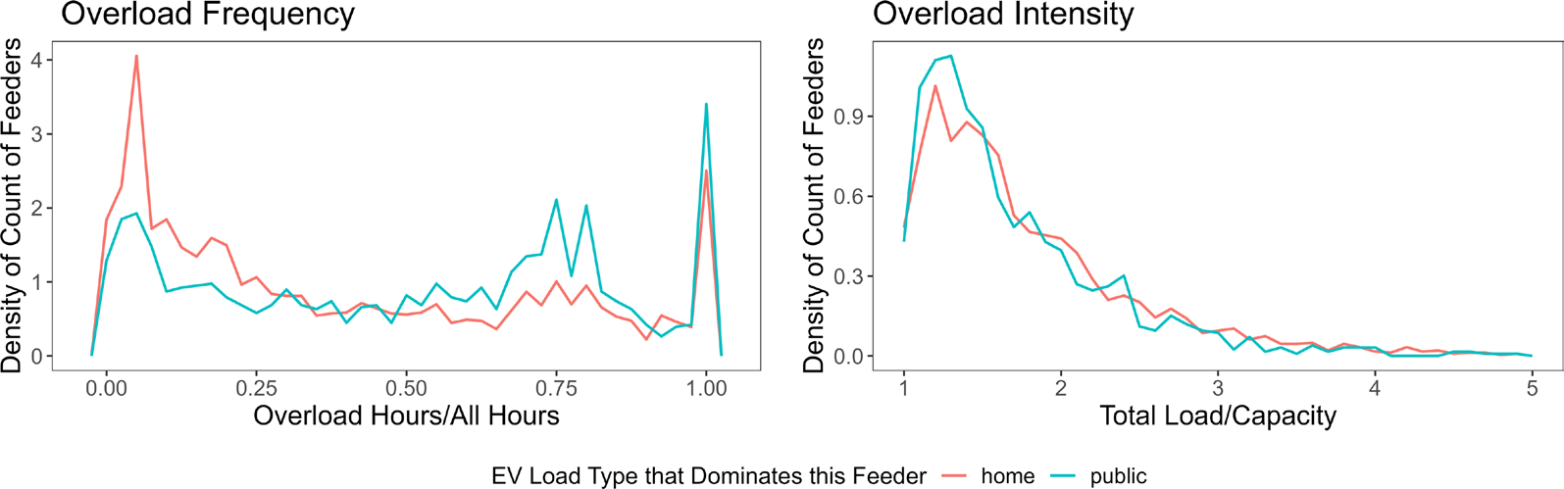
Density distribution of overload intensity (*Right*) and frequency (*Left*) of different feeder types in 2045. Overloading on home charging feeder is generally more intense and less frequent, while overloading on public-charging feeder tends to be less intense but more frequent. The distributions have been proven to be different from each other with the Kolmogorov–Smirnov test.

The substantial difference at the aggregate level is caused by nuances in the overload mechanism between home- and public-charging feeders. We examine the overload intensity with the ratio of total load over feeder capacity, and evaluate the overload frequency with the proportion of number of overload hours within all hours. The distributions of these two indices within home-charging feeders and public-charging feeders respectively are shown in Fig. 6. Home-charging feeders tend to have more intense but less frequent overloading, and public-charging feeders generally have less intense but more frequent overloading. This is related to the difference in both spatial and temporal distributions of home-charging versus public-charging demand. Spatially, 57.8% of the feeders are dominated by home-charging load, while the amount of home-charging energy takes up only 44.4% of the total charging demand. This means that home-charging load is more spatially clustered than public-charging load, even though they have the similar amount of total demand. Temporally, home-charging behaviors are usually clustered in the middle of the night, while public charging tends to be more spread out throughout the day. This difference in overload hours will be discussed further in detail in the next section. The temporal and spatial clustering of home-charging demand indicates the potential of reducing capacity upgrade costs by shifting the home-charging load, which will be discussed further in *Discussion and Conclusions*.

### Timing of Overloads.

**Fig. 7.**
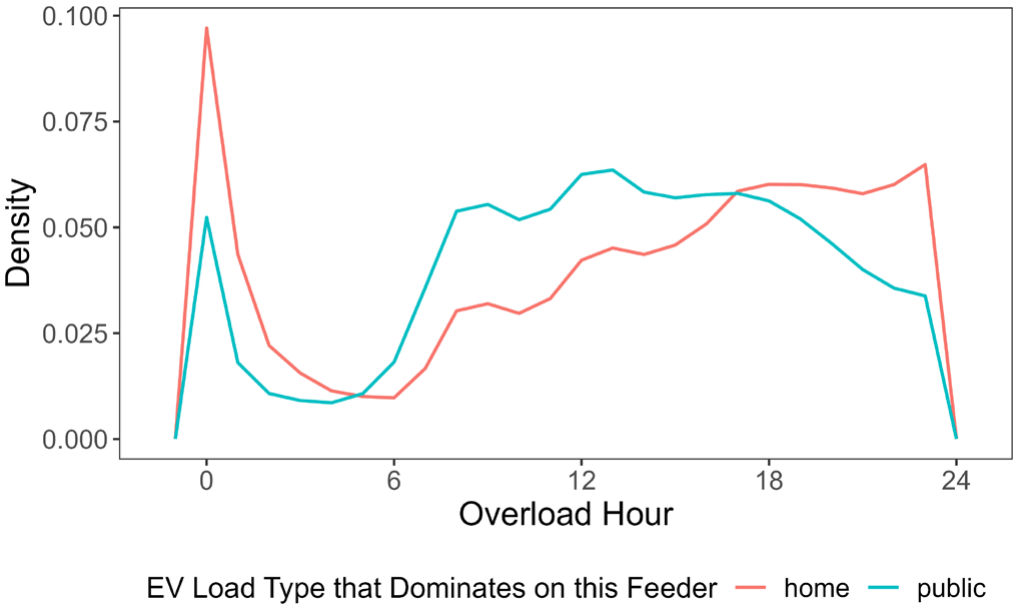
Overload hour distribution of different feeder types in 2045. On home charging feeders, majority of the overload take place during the night; while overloading on public-charging feeders happens more in the day.

The specific hours of the day that overload tends to occur varies across different charging locations. Fig. 7 shows the probability density distribution of overload hours on home charging–dominated feeders versus public charging–dominated feeders. On the feeders that have more home-charging demand, overload tends to happen more after 18:00, and overloading is most likely to occur at midnight; In contrast, for feeders that have more public-charging load, overloading is more likely to take place during the day than in the night, and the highest possibility for overloading occurs in late afternoon.

**Fig. 8.**
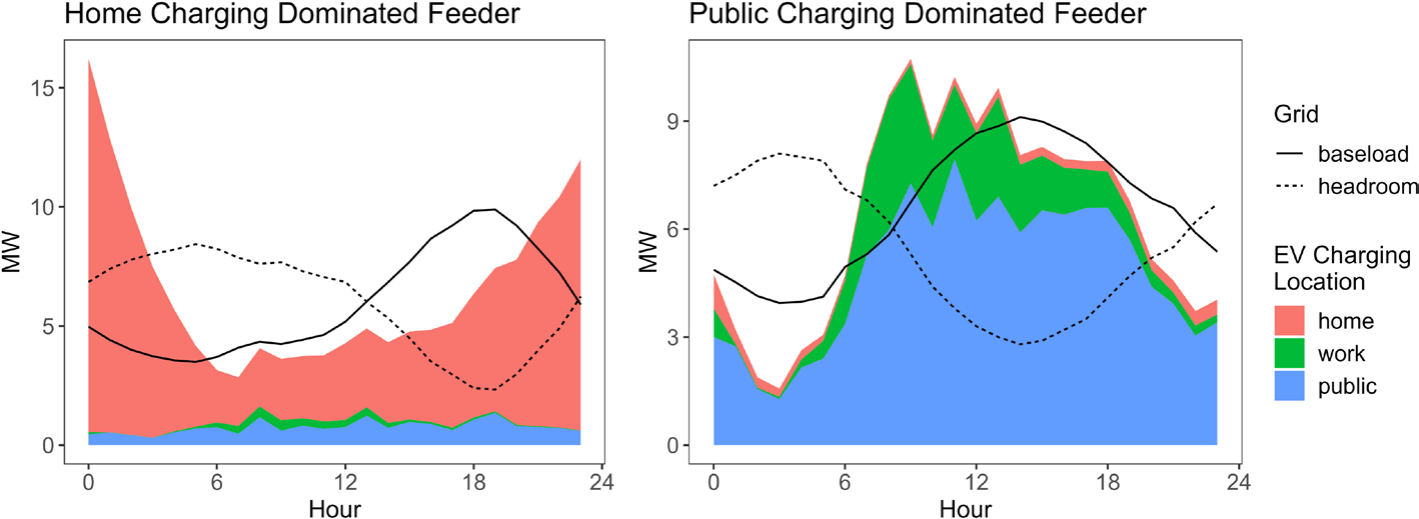
Feeder load pattern case study. Breakdown of hourly EV charging load by location, feeder baseload profile, and feeder headroom (remaining capacity excluding baseload), on a typical home charging–dominated feeder (*Left*) and a public charging–dominated feeder (*Right*), on the peak load day of August in 2045.

The hour that a feeder overload is a result of the synergy between baseload and EV load patterns. In Fig. 8 we present two case studies on the peak load day of August 2045 to explain this synergy. The graph on the left shows a typical feeder where the majority of EV charging load comes from home charging. The aggregate charging load pattern on this feeder has a high peak during the night and valley during the day. On the right, there is a typical public charging–dominated feeder, which is coupled with some workplace charging demand as well. It shows a cumulative charging pattern that peaks during the day. The composition of the EV charging load type is the result of local travel demand, which reflects the local facilities and type of buildings available, and is related to the land use or zoning of this area. The home charging–dominated feeder is likely located in a residential area, which explains its baseload pattern that has a peak in the evening around 18:00. The public charging–dominated feeder, on the other hand, is possibly from a commercial area, where the baseload peak occurs in the early afternoon. The combination of baseload and EV load pattern determines the time that overload tends to happen. On the residential feeder, overload happens during the night; and on the commercial feeder, most of the overload take place during the day.

Despite the general trend of difference discussed above, it is worth noting that the overloading time in each specific distribution network is more complicated than a residential versus commercial categorization. Most of the feeders in our analysis are a mix of home, public, and workplace charging load, so to determine the possible overloading hours, the local electricity consumer composition needs to be examined carefully.

### Sensitivity Analysis.

To test the sensitivity of our results to some key parameters and assumptions, we run the model under several extreme scenarios, results of which can be seen in Table 1. Improving EV energy efficiency from their current average efficiency to the highest observed efficiency can decrease the total capacity upgrade needs by 12%, while at the current lowest observed EV efficiencies, the total upgrade requirements would increase by 17%. Spatial shift of charging loads has the potential to reduce capacity upgrade needs. Allocating more charging events to the locations with more feeder capacity headroom left can reduce infrastructure upgrade costs by around 10%. And reducing the probability of home charging from 86 to 56% has a similar effect.

**Table 1.** Results under different scenarios in 2045

Scenario	Fraction of overloaded feeders	Required capacity upgrade (GW)	Total upgrade cost ($B) (25th, median, 75th percentile)
Baseline^*^	66.7%	25.4	6.2, 11.4, 20.0
EV efficiency 0.22 kWh/mile	64.4%	22.3	5.4, 10.3, 18.4
EV efficiency 0.47 kWh/mile	69.6%	29.8	7.2, 12.9, 22.5
0% DC fast charging in public charging	59.0%	19.6	4.8, 9.2, 16.5
100% DC fast charging in public charging	76.6%	53.3	12.8, 21.1, 35.1
Only single-family households (56%) have home-charging access	63.7%	22.1	5.4, 10.2, 18.3
Charging events allocated by feeder capacity headroom left	63.9%	23.3	5.7, 10.6, 18.8

^*^Baseline assumptions: EV efficiency 0.33 kWh/mile, 23% DC fast charging in public charging, 86% households have home-charging access, charging events allocated by population (for home charging) or number of jobs (for public and workplace charging) from TAZ to block level.

Overall, the sensitivity of our results remains within 20% of our baseline analysis under most of the scenarios, except for the extreme cases regarding DC fast charging. Removing all DC fast charging would result in a 12% drop in total number of feeders that need upgrade, and a 23% decrease in total capacity upgrade needs. On the other hand, if all public-charging events are conducted by DC fast charging, while the number of feeders that needs to be upgraded is only increased by 15%, the size of upgrade needs more than doubles due to the higher charging power demands. This implies that the adoption and usage of DC fast chargers need to be planned and managed carefully.

## Discussion and Conclusions

In this study, we utilize spatial and temporal data from both the grid and the EV side, to investigate to what extent the electric distribution system of California will be stressed by the growing EV charging load. We cover the spatial heterogeneity of circuit capacity and load profile by performing high-resolution analysis at the feeder level on a broad range of over 5,000 feeders statewide. The simulation of EV charging profile is conducted uniquely combining travel demand model, EV adoption model, and empirical EV charging data, with the EV uptake projections reflecting California’s ambitious policies.

On the aggregate level, we estimate that by 2035, 50% of the feeders in California will be overloaded by EV charging demand, and this percentage will grow to 67% by 2045. We project a total of 25 GW of circuit capacity upgrade needed by 2045, with a corresponding cost ranging from $6 billion to $20 billion, which is around 10 to 40% of the total existing distribution grid capital costs in the same territories. While the additional infrastructure cost drives the electricity price up, the growth of total electricity consumption drives the rate down, which leads to a net impact of rate reduction according to our estimation.

Feeders in residential areas are generally more stressed than those in commercial areas, with about twice the upgrade needed cumulatively. This is due to the spatial and temporal clustering of the home-charging demand compared to public and workplace charging demand, which contributes to more intense overloading in residential areas than in commercial areas. Although public charging sometimes has a much higher power level due to DC fast charging, the overloading intensity in commercial area feeders is dispersed by the spatial and temporal spread of charging demand. This indicates the potential of reducing grid infrastructure upgrade costs in residential areas by shifting home-charging load, both temporally flattening the charging load throughout the day, and spatially encouraging EV owners to relocate their charging activities from home to public or workplace. These potentials can be observed in our sensitivity analyses where reducing the probability of home charging from 86 to 56% can reduce infrastructure upgrade costs by around 10%.

Despite the general conclusions above, our results show a very high spatial diversity in the overload conditions across different feeders. The overloading intensity, frequency, and time are closely related to the local building types, which influence both EV charging behaviors and other electricity consumption behaviors. Other than that, when and how much overload takes place are also largely affected by the amount of capacity headroom left when the distribution infrastructure is built, as well as the overall scale of local EV charging load.

Moving forward, it is crucial to enact regulatory measures to accommodate and mitigate this expected infrastructure strain in the distribution network. In the short term, it is imperative to plan upgrades for the feeders that will experience highly frequent overloading. These feeders are expected to be overloaded almost all the time, which means that the upgrade caused by the general EV charging load growth is a necessity. For other feeders that are overloaded only at certain hours of each day, demand response mechanisms may be introduced to exploit the flexibility in EV charging behavior and reduce the significant peak load caused by the clustering of charging demand. For example, workplace and public charging could be encouraged by increasing charger availability at these locations, and/or differentiating charging rates based on location. EV owners’ participation in centralized charging control should be enabled and incentivized, so that overnight home-charging curves can be flattened.

This study offers insight into distribution grid planning, and provides a framework for evaluating EVs’ impact on the distribution grid. However, our findings should be interpreted in light of several limitations in the modeling framework. On the EV side, this study only considers the electrification of light-duty vehicles. Future work should address the impact of heavy-duty EVs, considering their potentially higher charging power that may stress the local distribution grid even more dramatically. On the grid side, possible future developments on the demand side are not yet included into the analysis, such as future demand growth, rooftop solar installation, and energy efficiency programs. On the EV-grid interaction side, it is worth noting that our analysis does not consider the influence of infrastructure availability on EV charging behavior. The decision of where in the distribution network gets to be upgraded first might gear the local EV adoption and travel demand.

## Materials and Methods

### Distribution Grid Data.

In 2016, the CPUC required utilities to perform ICA for the distribution system. The ICA maps from PG&E, SCE, and SDG&E provide publicly available data on the information of the distribution network down to the circuit level, including network pattern, feeder capacities, as well as hourly load profiles per feeder. The ICA data contains both thermal and voltage load allowances for their distribution circuit segments across each of the three major utilities in California. These capacities are calculated by running iterative steady-state power flow simulations at each node in the distribution system model: after adding existing baseload, incremental load is added per iteration to determine the maximum amount of load that can be penetrated without triggering thermal or voltage criteria violations (40). These load allowances are provided on an hourly basis by the maximum and minimum load days observed in each month of the year. Due to data availability issues of SCE, we use Grid Need Assessment (GNA) data for the feeder capacities in their territory. Unlike ICA data that is resolved by hour, the GNA data provides a single capacity rating value for each feeder, which is obtained by performing power system analysis on a single annual loading peak, and does not account for fluctuations from hour to hour (41). In our analysis, we adopt the ICA data in baseline year 2022, on the peak load day of each month (that is, the day with the highest baseload), when overload is most likely to happen. We then add the projected EV charging load to the baseload on each feeder to check whether the total load exceeds the feeder capacity, and the extent to which they are exceeded at each hour. The maximum overload observed on each feeder within each year is considered as the capacity upgrade need of this feeder.

PG&E provides data on their estimates of distribution grid upgrade projects and the corresponding investments in their Distribution Investment Deferral Framework (DIDF) map. Elmallah et al. (31) calculated the per-kW upgrade cost of each project, and divided these data into different scales to address the economies of scale (cost per-kW decrease with the increase of project scale). The authors use the 25th, 50th, and 75th percentiles of the per-kW costs within each scale group as references to calculate future grid upgrade costs. We apply the same methodology to calculate expected feeder upgrade costs according to our results of projected feeder capacity upgrade needs.

### EV Trip Projection.

We use the CSTDM together with the EV Toolbox (42) to determine the trips that are made by EVs. CSTDM simulates all the trips that take place in California in one typical weekday, including the origin and destination traffic analysis zones (TAZs), the distance of each trip, the household that this traveler belongs to, which TAZ is the household in, as well as the purpose of each trip (whether it is going home, to work, or to other public use locations like dining, shopping, school, recreation, etc.). To determine which of these trips are made by EVs, we adopt the EV Toolbox to project future EV adoption until 2045. The EV Toolbox uses diffusion of innovations model to estimate the number of households that will own at least one EV in the future in each census tract, considering social-economic factors of the households and retirement of existing vehicles (37, 43). With the share of EV households in each area, we are able to sample the households in CSTDM that are expected to own EVs at each year and determine the EV trips in each zone from CSTDM considering spatial heterogeneity.

We calibrate the model to ensure that EV sales growth aligns with the Zero-Emission Vehicle standard in California (2), which requires up to 100% EVs within all new light-duty vehicles sold in 2035. With this assumption, we project a state-wide EV adoption of around 6.6 million by 2030—which is more than 30% above the 5 million goal to be reached by 2030 and over 24 million EVs on the road by 2045.

### EV Charging Load Simulation.

To simulate EV charging load by feeder, we need to determine where the EVs will choose to charge during the EV trips. CSTDM classifies the trips into short- distance (generally below 300 miles) and long-distance (up to 700 miles), and we analyze these two classes separately.

For short-distance trips, CSTDM provides the information of whether several trips belong to a tour. So we assume that people would charge after a certain trip in each tour, but not necessary after each trip in a tour. We categorize the trip purposes into home, public, and workplace, as the main types of charging location. Then we bootstrap people’s choice of charging location from the distribution provided by the eVMT survey (39), which involves 7,979 EV owners and gives the proportion of households that choose different combinations of charging locations (only charge at home, charge at workplace or public, charge at all three types of locations, etc.).

For long-distance trips, we assume that people might charge in the middle of a trip around every 100 miles. Given the origin and destination of each trip, the trip route can be estimated based on major corridors with Djikstra’s algorithm. We then assume that public charging would take place at the intersection of the routes, and the charging type at destination is determined by the purpose of the whole trip. We are then able to determine the locations of the EV charging events for both short and long-distance trips. The charging demand of each event equals the corresponding split of travel distance within the tour or trip.

Next, we allocate each charging event to feeders throughout the state by disaggregating charging events at the TAZ level to a census block level, so that each zone is small enough to be mapped to only one feeder. For home-charging events, the spatial disaggregation is based on population (44) while public and workplace charging events are based on the number of jobs (45).

Finally, we generate the charging profile for each charging event using bootstrap simulation. The empirical EV charging data that we use include a total of 6,458,576 charging session records from: 1) charging network providers including EVgo, BTC POWER, and ChargePoint; 2) charging infrastructure installed by utilities; 3) the eVMT logger data, which is collected by installing data loggers on a total of 300 EVs in multiple utility areas, to track their travel and charging behaviors (46). The records range from 2011 to 2023 and include information of the start and end time of each charging session, as well as the charger level and charger location. More information of the datasets can be found in Table 2. We first divide the datasets into pools of home, public, and workplace charging based on the charging locations. Then, for each charging event that we derived from CSTDM, we calculate the energy consumption corresponding to its travel distance assuming an efficiency of 3 miles/kWh (47). The energy consumption of the trip, however, is not necessarily the exact amount of energy that an EV end up charging during the following charging event—it is possible that the battery is not full at the beginning of the trip, causing the EV to charge more than the energy consumed during the trip; or that the parking time is not long enough, causing the EV to charge less. To cover this uncertainty, we divide both the CSTDM trips and the empirical charging sessions into multiple bins of charging energy: 0 to 5, 5 to 10, 10 to 15, 15 to 20, 20 to 30, 30 to 50, 50 to 80, and above 80 kWh. For each charging event that we derived from CSTDM, we bootstrap a charging session from the corresponding trip purpose/charging location pool (home, work, public) and energy bin in the empirical dataset. Weighted bootstrap is conducted for public L2 vs DC charging—based on the numbers of L2 and DC fast chargers (48); and multifamily home vs single-family home-charging—based on the numbers of single-family units and multifamily units in California (44). Based on the spatial distribution of trip purposes and travel distances given by the CSTDM, we generate the specific charging time, energy, and power level of each charging event from the empirical data. Finally, we add up the charging power of all the charging events on each feeder by hour and obtain EV charging profile by feeder.

**Table 2.** Descriptive statistics of the empirical charging datasets used for generating charging profiles

Data source	Time period of collection	Charger level	Count of sessions
EVgo	2014–2019	Level 2	103,672
		DC	4,099,606
BTC power	2020–2022	DC	31,026
ChargePoint	2016–2022	DC	6,710
PG&E (EV charge network program)	2018–2022	Level 2	109,527
SCE (charge ready program)	2017–2022	Level 2	945,969
SDG&E (power your drive)	2017–2022	Level 2	812,628
SF city	2011–2023	Level 1	21,899
		Level 2	303,993
eVMT logger data (home charging)	2015–2020	Level 1	6,628
		Level 2	16,900

### Sensitivity Analysis.

We test the sensitivity of our results under several extreme scenarios. For the EV energy efficiency, we test the cases where all EVs are assumed to have the highest (0.22 kWh/mile) or lowest (0.47 kWh/mile) efficiency in the current battery EV models (47). To test the impact of varying charger power, we test the extreme cases of 0 and 100% DC fast charging within public charging by adjusting the weight in bootstrapping when sampling charging profiles from empirical charging records.

As described in the previous section, in the baseline scenario, people’s choice of charging location is derived from the eVMT survey, which reveals that 86% of the households would charge at home (39). However, it is possible that as EV adoption ramp up in the future, the proportion of households that performs home charging would decrease, as more and more consumers without access to home charging choose purchase EV. To simulate the other end of the spectrum, we test the scenario where the proportion of households that would choose home charging is equal to the share of single family units (56%) in California (44), while keeping the relative ratios of the other charging choices the same as eVMT survey results. This scenario also tests the potential effect of shifting home charging to public and workplace charging. Another scenario that tests the sensitivity to charging location is by changing the method of allocating charging events from TAZ level to block level. Instead of allocating by the ratio of population or number of jobs, we allocate the events by the ratio of feeder capacity headroom left in each block.

## Data Availability

The empirical EV charging data are available from the eVMT logger data, public-charging service providers, and utilities, but are not publicly available due to privacy concerns. Please direct charging data requests to Alan Jenn (ajenn@ucdavis.edu) and Gil Tal (gtal@ucdavis.edu). The distribution grid data are available from the ICA maps (PG&E: https://www.pge.com/en/about/doing-business-with-pge/interconnections/distributed-resource-planning-data-and-maps.html (49); SCE: https://drpep.sce.com/drpep/ (50); SDG&E: https://www.sdge.com/interconnection-information-and-map (51). The total electricity consumption (for the calculation of rate impact) and number of customers are available from the Energy Data Request Program (PG&E: https://pge-energydatarequest.com/public_datasets/download?type=electric&file=PGE_2022_Q4_ElectricUsageByZip.zip (52); SCE: https://www.sce.com/regulatory/energy-data---reports-and-compliances (53); SDG&E: https://energydata.sdge.com/ (54)). All original code has been deposited on GitHub from https://github.com/Yanningli2333/Distribution-Grid-EV-CA (55).
